# Draft Genome Sequence of a Fermenting Bacterium, “*Sphaerochaeta halotolerans*” 4-11^T^, from a Low-Temperature Petroleum Reservoir in Russia

**DOI:** 10.1128/MRA.01345-18

**Published:** 2018-11-29

**Authors:** Denis S. Grouzdev, Salimat K. Bidzhieva, Diyana S. Sokolova, Tatiyana P. Tourova, Ekaterina O. Patutina, Andrey B. Poltaraus, Tamara N. Nazina

**Affiliations:** a Institute of Bioengineering, Research Center of Biotechnology, Russian Academy of Sciences, Moscow, Russian Federation; b Winogradsky Institute of Microbiology, Research Center of Biotechnology, Russian Academy of Sciences, Moscow, Russian Federation; c Engelhardt Institute of Molecular Biology, Russian Academy of Sciences, Moscow, Russian Federation; University of California, Riverside

## Abstract

The draft genome sequence of an anaerobic fermenting bacterium, “*Sphaerochaeta halotolerans*” strain 4-11^T^, isolated from formation water of a low-temperature petroleum reservoir in Russia is presented. The genome is annotated to elucidate the taxonomic position of the strain 4-11^T^ and to extend the public genome database.

## ANNOUNCEMENT

The genus Sphaerochaeta of the family Spirochaetaceae comprises free-living anaerobic, mesophilic, neutrophilic, nonmotile bacteria, usually coccoid in shape, with fermentative metabolism (1). Four species are presently known, including Sphaerochaeta globosa, Sphaerochaeta pleomorpha, Sphaerochaeta coccoides (previously Spirochaeta coccoides), and Sphaerochaeta associata (2–4). Members of the genus Sphaerochaeta were isolated from freshwater sediments, methanogenic enrichments, wastewater sludge, and other anoxic habitats. Few pure cultures of the family Spirochaetaceae, namely Sediminispirochaeta smaragdinae (previously Spirochaeta smaragdinae) and Pleomorphochaeta caudata, have been isolated from oilfields (1, 5, 6).

Strain 4-11^T^ (=VKM B-3269) belonging to the genus Sphaerochaeta was isolated from production water of a low-temperature Vostochno-Anzirskoe oilfield in Russia (7). The strain was isolated in a peptone-glucose medium supplemented with NaCl to the final concentration of 40 g/liter at 35°C. Bacterial cells were nonmotile, spherical, ovoid, and pleomorphic with a diameter of 1 to 3 µm. The strain was an anaerobic chemoorganoheterotroph using a wide range of monosaccharides, disaccharides, and trisaccharides as carbon and energy sources. Strain 4-11^T^ grew optimally at 35°C, pH 6.0 to 6.5, and 10 g/liter NaCl. Phylogenetic analyses based on the 16S rRNA gene sequences showed that strain 4-11^T^ formed an independent branch within the genus Sphaerochaeta (7), sharing 96.8% and 96.4% similarity, respectively, with the genes of most closely related type strains S. associata GLS2^T^ and S. globosa Buddy^T^. In order to determine the taxonomic position of the new strain, its genome was sequenced and annotated.

For the isolation of genomic DNA, strain 4-11^T^ was anaerobically cultivated in a peptone-glucose medium supplemented with 4% (wt/vol) NaCl (7) at 35°C. Cells were harvested from 2 liter culture medium by centrifugation after 7 days of incubation, and the cell pellet was stored frozen (−20°C) until DNA preparation. Genomic DNA was extracted according to the method of Wilson (8), with minor modifications. Libraries were constructed with the NEBNext DNA library prep reagent set for Illumina, according to the protocol for the kit. Sequencing was carried out using the Illumina HiSeq 1500 platform with 250-bp single-end reads. Raw reads were quality checked with FastQC v.11.7 (https://www.bioinformatics.babraham.ac.uk/projects/fastqc/), and low-quality reads were trimmed using Trimmomatic v. 0.36 (9). Subsequently, the quality-filtered reads were *de novo* assembled with SPAdes v. 3.11.0 using the default settings (10).

A total of 1,393,290 reads were assembled into 49 contigs. This represented a 119× average coverage of the total sequence length of 2,927,075 bp. The largest contig was 334,155 bp, with an *N*_50_ value of 187,017 bp and a G+C content of 46.7%. Annotations of the contigs were carried out using the NCBI Prokaryotic Genome Annotation Pipeline (PGAP) (11).

The draft genome sequence of strain 4-11^T^ contained 2,788 genes, of which 2,698 were coding sequences, 34 were pseudogenes, and 45 were coded tRNAs. The 16S rRNA gene sequence revealed that the genome was identical to that determined earlier for the strain (7). The genome contained the genes associated with fermentation and carbohydrate metabolism. The average nucleotide identities (ANI) between strain 4-11^T^ and S. associata GLS2^T^ and S. globosa Buddy^T^ were 76.6% and 76.1%, respectively, and were below the <95% to 96% cutoff, which is generally accepted for species differentiation (12). Thus, the draft genome sequence of strain 4-11^T^ provides information about its affiliation with novel species within the genus Sphaerochaeta, with the proposed name “*Sphaerochaeta halotolerans*.”

### Data availability.

This whole-genome shotgun project has been deposited at DDBJ/ENA/GenBank under the accession no. QUWK00000000. The version described in this paper is the first version, QUWK01000000. The raw FASTQ reads have been deposited in the NCBI SRA database under the run no. SRR8109312.
